# Protocatechuic acid attenuated inflammation caused by *Prevotella copri* and its metabolites

**DOI:** 10.1080/21505594.2025.2609387

**Published:** 2025-12-23

**Authors:** Jiatai Gong, Siqi Ma, Hongkun Xiang, Xi Yang, Wentao Zhang, Ruizhi Hu, Ming Liu, Zhiyong Fan, Jianhua He, Shusong Wu

**Affiliations:** a Yuelushan Laboritory, Hunan Agricultural University, Changsha, China; b Animal Science and Technology College, Beijing University of Agriculture, Beijing, China

**Keywords:** *Prevotella copri*, protocatechuic acid, inflammation, metabolites, gut health

## Abstract

Gut microbiota has been considered as a key bridge between phytochemicals and host immunity. *Prevotella copri* (*P. copri)* showed a close correlation with inflammation, and protocatechuic acid (PCA) has potential protective effects in our previous studies. To understand the underlying mechanism, a total of 108 healthy Duroc × Landrace × Yorkshire weaned piglets, aged 21 d, were randomly assigned into 3 groups, with 6 replicates and 6 piglets per replicate. The piglets were fed a basal diet, a basal diet containing 1.0 × 10^8^ CFU/kg *P. copri* or 1.0 × 10^8^ CFU/kg *P. copri* +400 mg/kg PCA for 28 d. Results showed that *P. copri* decreased the final body weight and average daily gain (ADG), while increased the feed-to-gain ratio (F/G), with increased serum levels of interleukin (IL)-2 and IL-8 in piglets (*p < 0.05*), and reduced the expression of intestinal tight junction protein (*p < 0.05*). Dietary supplementation of PCA increased the ADG by suppressing inflammation and enhancing intestinal integrity. In vitro experiments demonstrated that argininosuccinic acid, indole-3-aldehyde, and N-acetylputrescine are critical metabolites produced by *P. copri*, which initiated inflammatory responses by upregulating pro-inflammatory cytokines and downregulating tight junction proteins in MODE-K cells. PCA was found to effectively attenuate these effects in a dose-dependent manner. In conclusion, PCA can improve the growth performance in weaned piglets by attenuating inflammation caused by *P. copri* and its metabolites.

## Introduction

The weaning phase in piglets is pivotal within the context of contemporary swine husbandry. The disorders of gut microbiota due to external influences, coupled with underdeveloped intestinal epithelium of piglets, are among the primary contributors to intestinal inflammation and diarrhea [1]. *Prevotella* constitutes a diverse genus of Gram-negative anaerobic bacteria prevalent in mammals [2], colonizing various regions of the body, including the skin, oral cavity, and gastrointestinal tract [3]. The functional role of *Prevotella* within the gut microbiota remains a subject of debate [4,5]. A decrease in *Prevotella* populations has been associated with the development of conditions such as Parkinson’s disease and autism [6]. Conversely, other studies have identified *Prevotella* as a potential pathogen, implicated in the initiation of diseases such as rheumatoid arthritis [7] and obesity [8]. *Prevotella* may augment immune responses [9,10]. The occurrence of these different situations is largely influenced by the host’s health and diet [9,11]. In weaned piglets, the onset of intestinal inflammation and diarrhea is characterized by a notable surge in the relative abundance of *Prevotella* within the intestinal tract [12], which in turn intensifies the inflammatory response [13]. Specific molecules exhibit a close interrelation. As a metabolically active microorganism, *Prevotella* is capable of producing a variety of bioactive metabolites, which function as key messengers in the initiation of host intestinal inflammation, including lipopolysaccharides (LPS) [14] and proteases [15,16]. These metabolites induce inflammation via the NF-κB signaling pathway and contribute to the degradation of the intestinal mucosa, compromising physical barriers and thereby increasing intestinal permeability and activating immune responses. Consequently, a systematic identification of the inflammatory metabolites produced by *P. copri*, along with an elucidation of its mechanisms of direct damage to the intestinal barrier, is essential for a comprehensive understanding of its pathogenicity.

PCA represents a prevalent class of natural phenolic acids within the plant kingdom [17]. Characterized by the presence of two hydroxyl groups, these compounds are commonly identified in various plant species, including raspberries, blueberries, mulberries, Eucommia ulmoides, and pine needles [18]. Functioning as primary phenolic metabolites of anthocyanins within the body [19], PCA exhibits notable antioxidant, antibacterial, and anti-inflammatory properties [20,21]. Recent studies have demonstrated that PCA can mitigate oxidative stress and inflammatory responses in piglets subjected to early weaning, thereby reducing the incidence of diarrhea associated with weaning stress, and improving growth performance [12,22].

Based on these results, the present study was designed to understand the mechanisms of PCA and *Prevotella* in modulating inflammation by using piglet and cell models.

## Materials and methods

### Ethics statement

The animal experimental protocol was approved by the Institutional Animal Care and Use Committee of Hunan Agricultural University (approval no: 2021056).

### Materials and agencies

PCA (≥98%) was provided by Shanghai Yuanye Bio-Technology Co., Ltd. (Shanghai, China). *P. copri* (DSM 18205) was purchased from the ATCC. The antibodies used in this experiment included Phospho-NF-κB p65 (Ser536) (#3033, Cell Signaling Technology), P65 (#6956, Cell Signaling Technology), Serum interleukin (IL)-1β (A7F7-R, HUABIO), IL-6 (BS62100, Bioworld), Cyclooxygenase-2 (COX2) (#12282, Cell Signaling Technology), Occludin (EPR20992, Abcam), Claudin-1 (EPR9306, Abcam), ZO-1 (HL1185, Abcam), β-actin (#4970, Cell Signaling Technology) (Table S1), and real-time fluorescence quantitative PCR primers (Table S2) were purchased from Sangon Biotech (Shanghai, China). The pigs used in the experiment were provided by Longhua Animal Husbandry Development Co., Ltd. in Hunan Province, China.

### Cultivation of *P. copri* and preparation of microbiological samples

*P. copri* is considered the most representative and widely prevalent strain within the *Prevotella* genus. The *P. copri* species are gram-negative anaerobic bacteria that require growth under anaerobic conditions at 37°C ^[3]^. Following subculturing, the bacterial population reached a stationary phase after 8 h. At this stage, the bacterial suspension was transferred from the culture bottle into a 50 mL centrifuge tube and centrifuged at 11,600 × *g* at 4°C for 10 min. Following centrifugation, we decanted the supernatant and subjected it to filtration using a 0.22 μm filter membrane to obtain a clean and sterile supernatant, which includes metabolites of *P. copri*. The sediment was the collected and freeze-dried to obtain it in powder form. Subsequently, the freeze-dried *P. copri* powder was carefully mixed with β-cyclodextrin at a concentration of 1.0 × 10^8^ CFU/g for use in piglet experiments. The remaining portion of the precipitate should be washed with sterile PBS and resuspended in PBS to achieve a concentration of 2.0 × 10^10^ CFU/mL. This suspension was then subjected to high-temperature sterilization (121°C, 20 min) and sonication to yield inactivated bacteria.

### Experimental design and diets

A total of 108 healthy Duroc × Landrace × Yorkshire crossbred piglets, weaned at 21 days of age, were randomly allocated into three experimental groups, each comprising six replicates with six piglets per replicate. The control group (CTL) was administered a basal diet formulated according to the National Research Council (NRC, 2012) guidelines (Table S2). The *P. copri* group (*P. copri*) received a diet supplemented with 1.0 × 10^8^ CFU/kg of *P. copri*, while the PCA group (PCA) was provided with a diet containing both 1.0 × 10^8^ CFU/kg of *P. copri* and 400 mg/kg of PCA for a duration of 28 days. In conducting the experiment on *P. copri* infection in weaned piglets and determining the dosage of PCA application, we drew upon the prior research findings [12,23]. Throughout the study, the piglets were housed in a pen equipped with a half-leaking floor and had unrestricted access to clean water and feed. The timeframe allocated for the execution of animal experiments spans from 25 July 2023, to 25 August 2023, encompassing both the preparatory phase and the formal testing procedures.

### Sample collections

Six piglets from each group, representing the average body weight of distinct replicate pens within each experimental group, were euthanized following anesthesia administration with sodium pentobarbital. The methodologies employed for the collection of blood and tissue samples can be referenced in the team’s prior study [24].

### Cell preparation and stimulation

The MODE-K cell line (UCSI373Mu11, Wuhan Cloud-Clone Corp., Ltd, China) was maintained at 37°C with 5% CO_2_ in DMEM medium (CellorLab, China) supplemented with 10% fetal bovine serum (FBS) (CellorLab, China) within an incubator. A passage operation was performed after the cells reached 70–80% confluency. Inoculate the specified cells into a 96-well plate. The control group was not treated and only replaced with blank culture medium. The experimental group mixed varying proportions of *P. copri* metabolites (supernatant) or inactivated bacteria with an empty culture medium at ratios of 1:1, 1:5, 1:10, 1:20, and 1:40 relative to the total volume. Incubate the treated cells for a duration of 24 h. Subsequently, the cells were incubated with a CCK-8 commercial kit (CellorLab, China) for 2 h. Absorbance was measured at 450 nm using a porous chemiluminescence analyzer (Varioskan Flash).

The cells were inoculated into 10 cm culture dishes and allowed to reach 70–80% confluence. The medium was then replaced with serum-free medium, and *P. copri* metabolites (supernatant) or heat-inactivated bacteria were added at a concentration equivalent to one-fifth of the total culture volume. Following treatment, cells were cultured for 24 h. Collected cell samples were used for Western blot analysis, with Brefeldin A added 3 h prior to sample collection. Based on this procedure, varying concentrations of PCA (0, 50, 100, and 200 µM/L) were added, and cells were incubated for an additional 24 h. Thereafter, cell samples were collected to assess the expression of inflammatory cytokines and tight junction proteins by Western blot analysis.

### Western blotting

The western blot was conducted as reported in our previous study [25]. Briefly, ileum samples were precisely weighed (less than 100 mg) and lysed with RIPA buffer at a mass-to-volume ratio of 1:9 for western blotting. For cellular samples, the culture medium was first removed, the cells were washed with PBS, and then 100 µL of RIPA lysis buffer was added per plate (Table S2).

### Measurement of antioxidant indices and inflammatory cytokines in serum

IL-2, IL-8 and IL-10 were quantified using enzyme-linked immunosorbent assay kits (Jianglai Bioengineering) according to their protocols.

### Real-time polymerase chain reaction (RT-PCR)

In this study, the mRNA expression levels of inflammatory cytokines and tight junctional protein in the ileum were quantified using real-time quantitative PCR. Total RNA was extracted from the samples using TRIzol Reagent (Sangon Biotech, Shanghai, China), following the manufacturer’s protocol. The experimental procedure for quantitative PCR will be conducted in accordance with the methodologies delineated in prior published studies [26]. β-actin served as an endogenous reference gene, with the specific gene primers detailed in Table S3.

### Immunofluorescence

The methodology for conducting immunofluorescence experiments has been described previously [12]. Briefly, following the preparation of tissue sections, they were sequentially incubated with primary and secondary antibodies. The stained sections were then examined using a Motic BA210T microscope (Motic China Group Co., Ltd., Xiamen, Fujian, China).

### Metabolomics by LC-MS

The bacterial metabolites in culture supernatant and inactivated bacteria were profiled by Shanghai Majorbio Co., Ltd. (China) using LC-MS according to a well-documented report [27]. The raw data were processed using Progenesis QI software (Waters, Milford, USA) to generate a data matrix. Metabolites were subsequently classified according to the Human Metabolome Database (http://www.hmdb.ca/), and enrichment analysis was performed utilizing the KEGG database (http://www.genome.jp/kegg/).

### Chemical analyses

The values for digestible energy (DE), net energy (NE), standardized ileal digestible (SID) amino acids, and digestible phosphorus (P) were calculated, while the remaining parameters were measured. The methodologies employed for the quantification of energy and nutrients were derived from the standards outlined by a previous study [23].

### Statistical analysis

The results are presented as the mean ± standard error of the mean (SEM). Statistical differences between groups were assessed using the t-test or one-way analysis of variance (ANOVA), followed by post hoc analyses with Fisher’s least significant difference (LSD) test and Duncan’s multiple range test. Principal component analysis (PCA) was employed to illustrate the differences and distances among samples. A p-value of less than 0.05 was considered statistically significant, while a p-value of less than 0.01 was considered highly significant.

## Results

### Effects of *P. copri* and PCA on the growth performance and inflammation in weaned piglets

The final weight and average daily weight gain (ADG) in the *P. copri* group were significantly lower compared to the other two groups, and the feed-to-gain ratio (F/G) was significantly higher than that of the control (CTL) group (*p < 0.05*). Following the supplementation of PCA, both the final weight and ADG were significantly greater than those observed in the *P. copri* group (Table 1, *p < 0.05*).

**Table 1.** Effects of *P. copri* and PCA on growth performance in weaned piglets.

	CTL	*P. copri*	PCA	SEM	*p*-value
Initial weight/kg	5.90	6.08	6.00	0.042	0.222
Final weight/kg	16.59^a^	15.31^b^	16.11^a^	0.189	0.017
ADG (g/day)	381.85^a^	336.64^c^	361.10^b^	6.623	< 0.01
ADFI (g/day)	508.87	493.56	512.09	4.677	0.246
F/G (g/g)	1.33 ^b^	1.47 ^a^	1.41 ^a^	0.021	< 0.01

ADG = average daily gain; ADFI = average daily feed intake; F/G = feed-to-gain ratio.

CTL = a basal diet; *P. copri* = a basal diet containing 1.0 × 10^8^ CFU/kg of *P. copri*; PCA = a basal diet supplemented with 1.0 × 10^8^ CFU/kg of *P. copri* and 400 mg/kg of Protocatechuic Acid (PCA).

Initial weight and final weight data were shown as means ± SEM (*n* = 30). ADG, ADFI, and F/G data were shown as mean ± SEM (*n* = 3).

^a, b, c^Values with different superscript letters differ significantly (*p < 0.05*).

**Figure 1.**
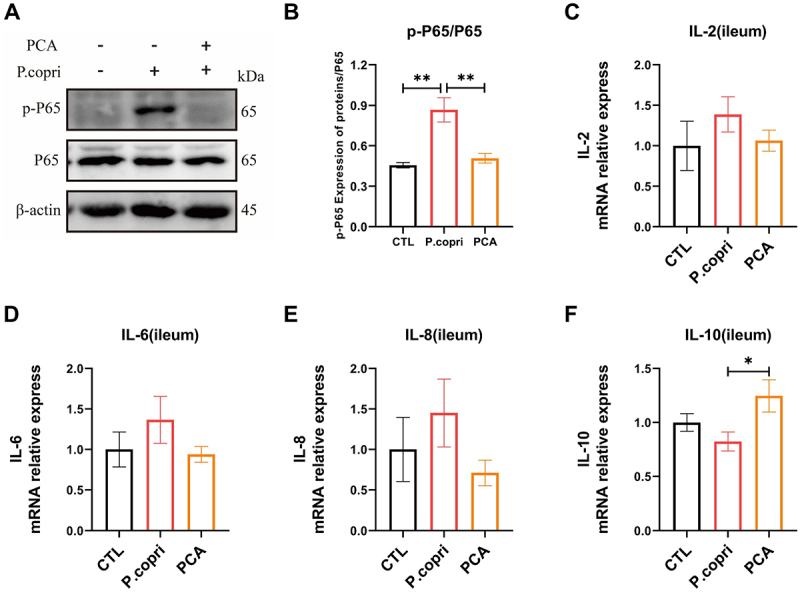
PCA alleviated intestinal inflammation in weaned piglets caused by *P. copri*. Analyze the phosphorylation level of P65 by Western blotting (*n* = 3) (A, B); real-time PCR was used to detect the mRNA expression levels of *interleukin (il)-2* (C), *IL-6* (D), *IL-8* (E), and *IL-10* (f) in ileal tissue (*n* = 6). Data are shown as the means ± SEMs, * *p < 0.05*, ** *p* < 0.01.

This study assessed the impact of *P. copri* on inflammation in weaned piglets and examined the regulatory effects of PCA on inflammatory responses. After 4 weeks, a significant increase in serum levels of IL-2, IL-8 was observed in the *P. copri* group (*p < 0.05*), accompanied by a reduction in IL-10 activity, although the difference was not statistically significant (*p* = 0.641) (Table 2). The phosphorylation status of the P65 protein in ileal tissue was assessed, and results demonstrated that *P. copri* significantly increased P65 phosphorylation (*p < 0.01*) (Figure 1(A,B)). Conversely, the inclusion of PCA significantly attenuated these effects (*p < 0.05*). A parallel trend was observed at the transcriptional level, where the mRNA expression of *IL-2*, *IL-6*, and *IL-8* was up-regulated in the *P. copri* group compared to the CTL group and down-regulated upon PCA treatment, albeit without reaching statistical significance (Figure 1(C-F)). In conclusion, the findings indicated that the consumption of *P. copri* adversely affected the growth performance of weaned piglets, increased the feed-to-weight ratio, and exacerbated inflammatory responses. Nevertheless, the application of PCA appeared to mitigate these negative effects.

**Table 2.** Effects of *P. copri* and PCA on serum inflammatory and antioxidant markers.

	CTL	*P. copri*	PCA	SEM	*p*-value
IL-2, ng/L	2467.348^b^	3134.239^a^	2490.062^b^	104.430	< 0.01
IL-8, ng/L	6896.564^b^	8911.840^a^	7123.057^b^	294.537	< 0.01
IL-10,ng/L	1156.057	1106.080	1187.942	34.031	0.641

IL-2 = interleukin-2; IL-8 = interleukin-8; IL-10 = interleukin-10.

CTL = a basal diet; P. copri = a basal diet containing 1.0 × 108 CFU/kg of P. copri; PCA = a basal diet supplemented with 1.0 × 108 CFU/kg of P. copri and 400 mg/kg of Protocatechuic Acid (PCA).

Data were shown as means ± SEM (*n* = 6). ^a, b^ Values with different superscript letters differ significantly (*p < 0.05*).

### Modulation of intestinal tight junction proteins by *P. copri* and PCA

**Figure 2.**
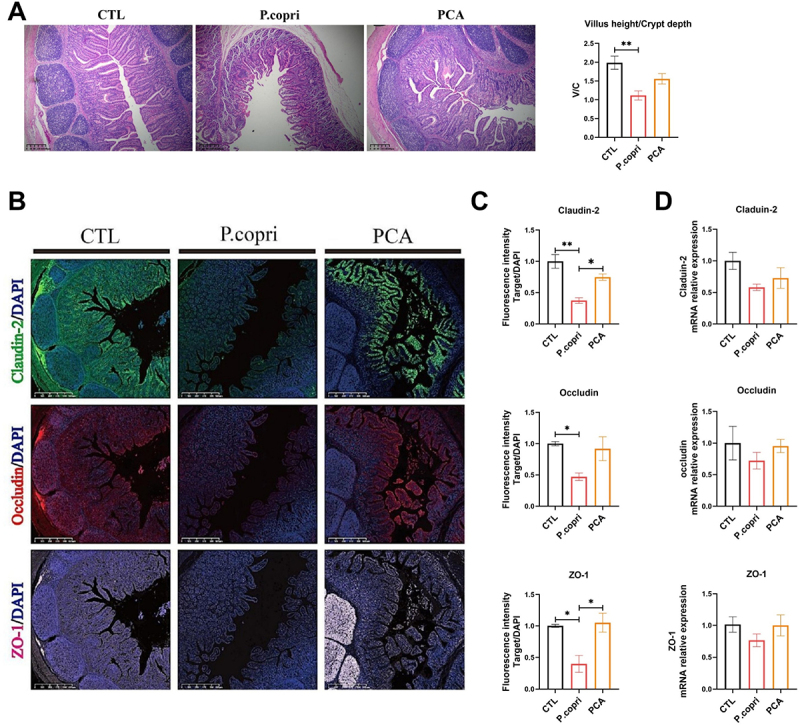
PCA increased intestinal tight junction protein expression. H&E stained colon sections and villus height/crypt depth ratio in ileal tissue (*n* = 6) (A); immunofluorescence staining of target [incluing Claudin-2, Occludin, Zonula Occludens (ZO)-1] in DAPI (B); the protein expression of claudin-2, Occludin, ZO-1 was detected by immunofluorescence (*n* = 3) (C); real time PCR was used to detect the mRNA expression levels of *Claudin-2*, *Occudin* and *ZO-1* (*n* = 6) (D); data are shown as the means ± SEMs, * *p < 0.05*, ** *p* < 0.01.

*P. copri* damaged the intestinal morphology and significantly reduced the ratio of intestinal villi height to crypt depth (*p < 0.01*) (Figure 2(A)). To examine the impact of *P. copri* and PCA on intestinal tight junction proteins, the study assessed the expression levels of Claudin-2, ZO-1, and Occludin in ileal tissue through immunofluorescence staining. As illustrated in Figure 2(B,C) the administration of *P. copri* significantly decreased the protein expression of Claudin-2, ZO-1, and Occludin, whereas supplementation with PCA effectively counteracted these effects (*p < 0.05*). In parallel, the mRNA expression of intestinal tissue showed the same tendency, though the changes were not statistically significant (Figure 2(D)).

### *P. copri* and its metabolites can both cause inflammation

**Figure 3.**
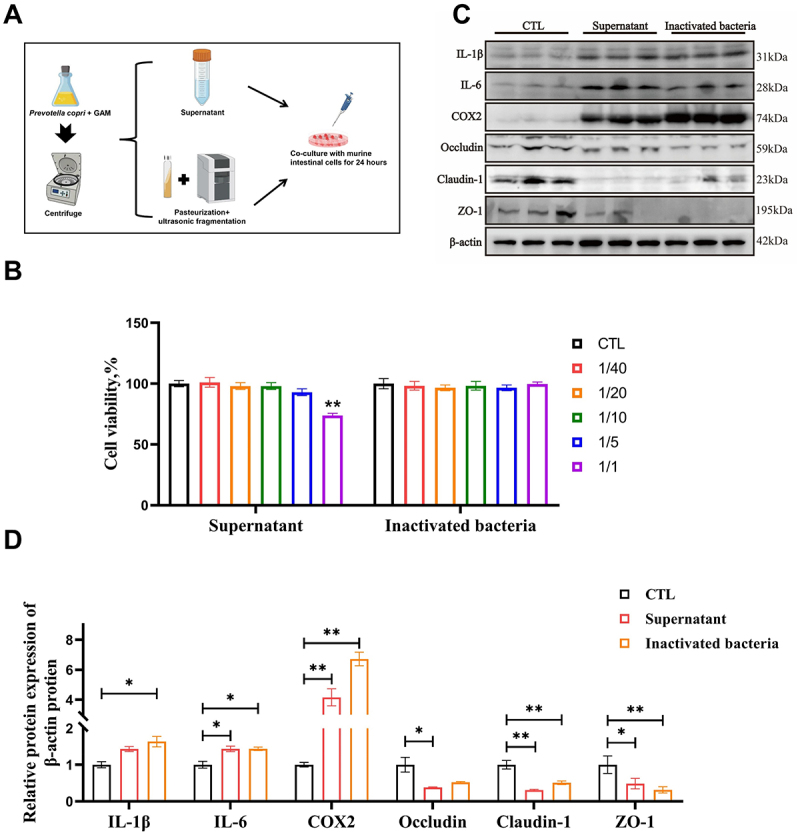
Metabolites and inactivated bacteria of *P. copri* can cause inflammation. Schematic of the experimental design (A); the impact of metabolites and inactivated bacteria on cell survival rate (*n* = 12) (B); the effects of mixing supernatant or inactivated bacteria with culture medium at a ratio of 1:5 on the protein expression levels of IL-1 β, IL-6, cyclooxygenase (COX) −2, Occludin, claudin-1, and ZO-1 in cells after treatment (*n* = 3) (C, D). Data are shown as the means ± SEMs, * *p < 0.05*, ** *p* < 0.01.

During the growth of *P. copri*, various metabolites are produced, and decomposition after cell death releases additional substances. The metabolites (culture medium supernatant), following the cultivation of *P. copri*, along with heat-inactivated bacteria, were collected and subsequently co-cultured with the MODE-K cells (Figure 3(A)). The results indicated that high concentrations of metabolites (supernatant: culture medium = 1:1) can reduce cell survival rate (Figure 3(B)). However, they did lead to a significant increase in the expression of inflammatory cytokines IL-1β and IL-6, as well as COX2. Additionally, the expression of tight junction proteins Occludin, Claudin-1, and ZO-1 was significantly reduced (*p < 0.05*) (Figure 3(C,D)).

### Unraveling potential virulence factor candidates in *P. copri*

**Figure 4.**
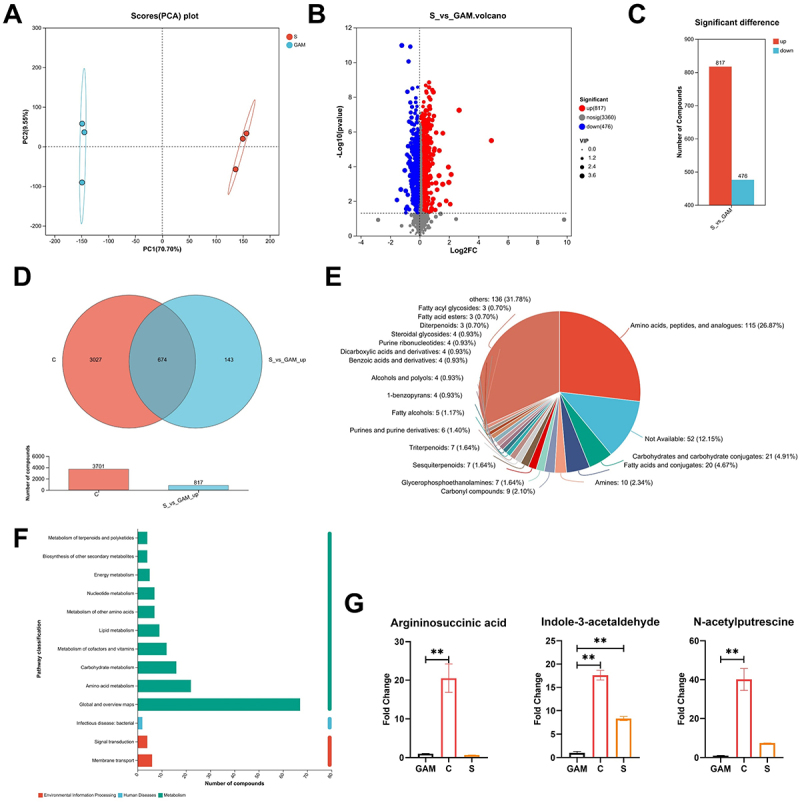
Identification of key metabolites secreted by *P. copri*. Cluster analysis by using PCA (A); S_vs_GAM differential metabolite volcano plot (B) and bar chart (C); venn diagram of differential metabolites (D); overlapping metabolites were classified by human metabolome database (HMDB) database (E); Kyoto encyclopedia of genes and genomes (KEGG) pathway enrichment analysis of overlapping expressed metabolites (F); the relative content proportion of signature metabolites (G). (note: S represents the supernatant; C represents the inactivated bacteria, and gam represents the blank medium). Data are shown as the means ± SEMs (*n* = 3), * *p < 0.05*, ** *p* < 0.01.

To elucidate the metabolites that regulate intestinal barrier function, the study conducted a non-targeted metabolomics analysis on the supernatant and inactivated bacteria from the *P. copri* culture medium. Initially, we compared the supernatant (S) of the *P. copri* culture with the blank culture medium (S_vs_GAM). Principal component analysis revealed a significant separation between these two groups (Figure 4(A)). Through this comparison, 817 up-regulated differential metabolites were identified (Figure 4(B,C)). A Venn analysis of these differential metabolites and the inactivated bacteria (C) revealed 674 overlapping metabolites (Figure 4(D)), predominantly comprising amino acids, peptides, and their analogues (Figure 4(E)). KEGG enrichment analysis indicated that these metabolites were primarily associated with the amino acid metabolism pathway (Figure 4(F)). Subsequently, the substances enriched in the amino acid metabolism pathway were analyzed one by one, among which argininosuccinic acid, indole-3-aldehyde, and N-acetylputrescine were the three substances that best matched the trend of phenotypic changes, that is, positively correlated with the occurrence of inflammation (Figure 4(G)).

### PCA can alleviate inflammation caused by metabolites and inactivated bacteria

**Figure 5.**
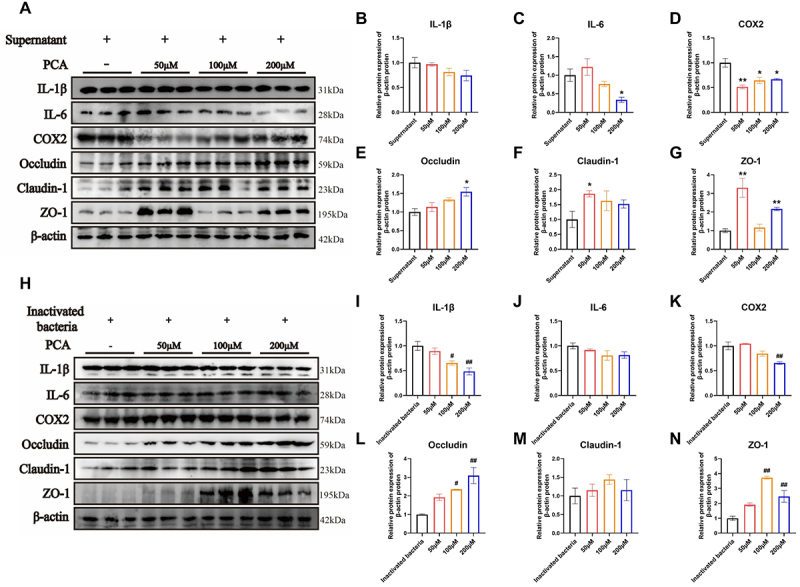
PCA can alleviate inflammation caused by metabolites and inactivated bacteria. The effect of *P. copri* metabolites on the expression of inflammatory cytokines and tight junction proteins (A-G); the effect of *P. copri* inactivated bacteria on the expression of inflammatory cytokines and tight junction proteins (H-N). Data are shown as the means ± SEMs (*n* = 3), * *p < 0.05*, ** *p* < 0.01 compared with supernatant. ^#^ *p < 0.05*, ^##^ *p* < 0.01 compared with inactivated bacteria.

In this study, the impact of PCA on diminishing the relative abundance of *g_Prevotella* was found to be limited (Fig. S1); however, it significantly mitigated inflammation induced by *P. copri*. Consequently, it is hypothesized that PCA may play a role in alleviating inflammation caused by *P. copri* metabolites and inactivated bacteria. To investigate this, varying concentrations of PCA were added alongside metabolites or inactivated bacteria into the culture medium, which was then co-cultured with cells. The expression levels of pro-inflammatory cytokines and tight junction proteins were subsequently assessed (Figure 5(A,H)). High concentration of PCA (200 μM) significantly decreased the expression levels of IL-6 in inflammation induced by metabolites; meanwhile, it significantly reduced the expression level of COX2 (*p < 0.05*) (Figure 5(B-D)). In the experimental group treated with inactivated bacteria, high concentrations of PCA led to a significant reduction in the expression of IL-1β and COX2 (*p < 0.01*) (Figure 5(I-K)). Notably, PCA was found to significantly enhance the expression of tight junction proteins (*p < 0.05*) (Figure 5(E-G,L-N)).

## Discussion

The weaning phase represents a critical and challenging period, during which piglets are susceptible to significant stress-related injuries, particularly concerning intestinal health. The substantial environmental and nutritional transitions associated with weaning can precipitate severe stress-induced intestinal damage, with the pathological processes intricately linked to disruptions in the microbial community. *Prevotella*, an opportunistic pathogen, is significantly influenced by the host’s health status. Studies have indicated that inflammation in weaned piglets correlates with a marked increase in the relative abundance of *Prevotella* within their intestines [28,29]. In this experiment, the role of *Prevotella* in inducing inflammation and enhancing intestinal permeability, as well as the mitigating effect of PCA, was confirmed by supplementing the diet of weaned piglets with *P. copri* and PCA.

In this study, it was observed that *P. copri* significantly upregulated the expression of inflammatory cytokines and transcription factors. The lipopolysaccharide produced by *P. copri* facilitates the release of inflammatory cytokines by upregulating the nuclear factor kappa B (NF-κB) signaling pathway [30]. Furthermore, research has demonstrated that *P. copri* can produce various endopeptidases, including serine proteases and cysteine enzymes, which play a significant role in evading host immune responses and modulating inflammatory disease-related signaling pathways [15,16]. In addition to the metabolites of *P. copri* that can induce inflammation, this study identified the presence of an inflammatory response through the co-culturing of inactivated *P. copri* with cells. Consequently, this study employed non-targeted metabolomics techniques to identify substances co-expressed by *P*. *copri* metabolites and bacterial cells, thereby identifying key mediators that induce inflammation. Argininosuccinic acid, indole-3-aldehyde, and N-acetylputrescine, which were significant pro-inflammatory compounds co-expressed by metabolites of *P. copri* and inactivated bacteria, have been demonstrated to exhibit a positive correlation with the pathological state of the organism [31–33], the upregulated expression of these markers underscores the significant role of *P. copri* in pro-inflammatory processes. Cell experiments revealed a marked decrease in the expression levels of intestinal tight junction proteins, which may be associated with the presence of enzymes in *P. copri* that are crucial for mucin degradation [34], *P. copri* expressed mucin and glycoside hydrolase, which reduced the mucin layer’s thickness and lowered tight junction protein expression, thereby increasing the intestinal permeability.

In conclusion, this study indicated that the promotion of intestinal inflammation by *P. copri* is a multifaceted and intricate process. Notably, our research had associated this pro-inflammatory effect with three specific metabolites identified through untargeted metabolomics: argininosuccinic acid, indole-3-aldehyde, and N-acetylputrescine. These findings offer a novel perspective for comprehending its pathogenic mechanism. Within the nitric oxide (NO) synthesis pathway, the aberrant accumulation of argininosuccinic acid may interfere with arginine metabolism in intestinal cells, leading to oxidative stress and inflammatory responses [35,36]. Indole-3-acetaldehyde, recognized as a ligand for the aromatic hydrocarbon receptor (AhR) [37], is implicated in the complex regulation of intestinal homeostasis; however, its dysregulated activation may provoke pro-inflammatory immune responses under certain conditions, aligning with the noted upregulation of pro-inflammatory cytokines [38]. As a byproduct of polyamine metabolism, abnormal levels of N-acetylputrescine may contribute to epithelial barrier dysfunction, potentially explaining the observed downregulation of tight junction proteins such as Occludin and ZO-1 [39,40]. Consequently, the pathogenicity of *P. copri* was attributed not only to its structural components, such as LPS, but also to the specific bioactive metabolites it secretes, which collectively compromise the immune and physical barriers of the intestine.

Polyphenols exhibit multiple biological functions [41], upon entering the gastrointestinal tract, PCA can influence the gut microbiota and concurrently inhibit the growth of pathogenic bacteria, including both Gram-positive and Gram-negative species [42]. PCA primarily exerts its anti-inflammatory effects by modulating the NF-κB and mitogen-activated protein kinase (MAPK) pathways [43,44], and it can inhibit the expression of COX2 [45], thereby decreasing the production of inflammatory cytokines. In this study, *P. copri* induced the phosphorylation activation of P65, leading to an increase in COX2; however, PCA alleviated the expression of IL-1β and IL-6 by reducing the phosphorylation level of p65. Some polyphenolic compounds are thought to interact with enzyme active sites, directly inhibiting enzyme activity [46,47]. While our data clearly demonstrated that PCA enhanced the expression of intestinal tight junction proteins, the upstream mechanisms may be multifaceted. One potential, albeit unverified, mechanism could involve the inhibition of *P. copri*-derived mucin-degrading enzymes by PCA, analogous to the documented enzyme-inhibitory effects of other polyphenolic compounds [48]. This hypothesis represents a promising direction for elucidating the full scope of PCA’s protective actions.

## Conclusion

This study demonstrated that dietary supplementation with *P. copri* induced intestinal inflammation and compromised barrier integrity in weaned piglets, thereby negatively impacting growth performance. More importantly, by employing a combination of *in vivo* and *in vitro* models, we identified that these detrimental effects were mediated not only by the bacterial cells themselves but also, crucially, by their secreted metabolites. We pinpointed three specific amino acid-derived metabolites – argininosuccinic acid, indole-3-aldehyde, and N-acetylputrescine – as key virulence factors contributing to the inflammatory response. Furthermore, our findings indicated that the natural polyphenol PCA significantly enhances intestinal barrier function by downregulating the expression of inflammatory mediators and upregulating the expression of tight junction proteins. Our findings elucidated a novel mechanism by which *P. copri* impairs gut health and provide evidence for PCA as a promising dietary intervention to counteract these effects.

## Supplementary Material

Supplementary data.docx

Author Checklist E10 only.pdf

## Data Availability

The data that support the findings of this study are openly available in Mendeley Data at https://data.mendeley.com/datasets/hzcfbrxmcg/1 [49], reference number “10.17632/hzcfbrxmcg.1.”

## References

[1] Ma X, Zhang Y, Xu T, et al. Early-life intervention using exogenous fecal microbiota alleviates gut injury and reduce inflammation caused by weaning stress in piglets [J]. Front Microbiol. 2021;12. doi: 10.3389/fmicb.2021.671683 PMC8222923 34177852

[2] Zhou N, Huang H, Liu H, et al. Microbiota analysis of peri-implant mucositis in patients with periodontitis history [J]. Clin Oral Investig. 2022;26(10):6223–12. doi: 10.1007/s00784-022-04571-1 PMC9525361 35672515

[3] Tett A, Pasolli E, Masetti G, et al. Prevotella diversity, niches and interactions with the human host [J]. Nat Rev Microbiol. 2021;19(9):585–599. doi: 10.1038/s41579-021-00559-y 34050328 PMC11290707

[4] Ley RE. Gut microbiota in 2015: prevotella in the gut: choose carefully [J]. Nat Rev Gastroenterol Hepatol. 2016;13(2):69–70. doi: 10.1038/nrgastro.2016.4 26828918

[5] Cani PD. Human gut microbiome: hopes, threats and promises [J]. Gut. 2018;67(9):1716–1725. doi: 10.1136/gutjnl-2018-316723 29934437 PMC6109275

[6] Kang D-W, Park JG, Ilhan ZE, et al. Reduced incidence of Prevotella and other fermenters in intestinal microflora of autistic children [J]. PLOS ONE. 2013;8(7):e68322. doi: 10.1371/journal.pone.0068322 23844187 PMC3700858

[7] Maeda Y, Kurakawa T, Umemoto E, et al. Dysbiosis contributes to arthritis development via activation of autoreactive T cells in the intestine [J]. Arthritis Rheumatol. 2016;68(11):2646–2661. doi: 10.1002/art.39783 27333153

[8] Gong J, Zhang Q, Hu R, et al. Effects of Prevotella copri on insulin, gut microbiota and bile acids [J]. Gut Microbes. 2024;16(1):2340487. doi: 10.1080/19490976.2024.2340487 38626129 PMC11028016

[9] Kovatcheva-Datchary P, Nilsson A, Akrami R, et al. Dietary fiber-induced improvement in glucose metabolism is associated with increased abundance of Prevotella [J]. Cell Metab. 2015;22(6):971–982. doi: 10.1016/j.cmet.2015.10.001 26552345

[10] Abdelsalam NA, Hegazy SM, Aziz RK. The curious case of Prevotella copri [J]. Gut Microbes. 2023;15(2):2249152. doi: 10.1080/19490976.2023.2249152 37655441 PMC10478744

[11] DE Vadder F, Kovatcheva-Datchary P, Zitoun C, et al. Microbiota-produced succinate improves glucose homeostasis via intestinal gluconeogenesis [J]. Cell Metab. 2016;24(1):151–157. doi: 10.1016/j.cmet.2016.06.013 27411015

[12] Hu R, He Z, Liu M, et al. Dietary protocatechuic acid ameliorates inflammation and up-regulates intestinal tight junction proteins by modulating gut microbiota in LPS-challenged piglets [J]. J Anim Sci Biotechnol. 2020;11(1). doi: 10.1186/s40104-020-00492-9 PMC7487840 32944233

[13] Chen C, Fang S, Wei H, et al. Prevotella copri increases fat accumulation in pigs fed with formula diets [J]. Microbiome. 2021;9(1):175. doi: 10.1186/s40168-021-01110-0 34419147 PMC8380364

[14] Lin T-L, Shu C-C, Chen Y-M, et al. Like cures like: pharmacological activity of anti-inflammatory lipopolysaccharides from gut microbiome [J]. Front Pharmacol. 2020;11. doi: 10.3389/fphar.2020.00554 PMC7212368 32425790

[15] Turk B. Targeting proteases: successes, failures and future prospects [J]. Nat Rev Drug Discov. 2006;5(9):785–799. doi: 10.1038/nrd2092 16955069

[16] Lopez-Otin C, Hunter T. The regulatory crosstalk between kinases and proteases in cancer [J]. Nat Rev Cancer. 2010;10(4):278–292. doi: 10.1038/nrc2823 20300104

[17] Rao Y, Wang J, Zhao R, et al. Efficient sustainable production of protocatechuic acid from glucose by engineered Bacillus licheniformis [J]. Chem Eng J. 2025;505:159320. doi: 10.1016/j.cej.2025.159320

[18] Kakkar S, Bais S. A review on protocatechuic acid and its pharmacological potential [J]. ISRN Pharmacol. 2014;2014(952943):1–9. doi: 10.1155/2014/952943 PMC4005030 25006494

[19] DE Ferrars RM, Czank C, Zhang Q, et al. The pharmacokinetics of anthocyanins and their metabolites in humans [J]. Br J Pharmacol. 2014;171(13):3268–3282. doi: 10.1111/bph.12676 24602005 PMC4080980

[20] Zhang S, Gai Z, Gui T, et al. Antioxidant effects of protocatechuic acid and protocatechuic aldehyde: old wine in a new bottle [J]. Evidence-Based Complementary Alternat Med. 2021;2021:1–19. doi: 10.1155/2021/6139308 PMC8592717 34790246

[21] Zhang J, Fu B, Chen X, et al. Protocatechuic acid attenuates anterior cruciate ligament transection-induced osteoarthritis by suppressing osteoclastogenesis [J]. Exp Ther Med. 2020;19(1):232–240. doi: 10.3892/etm.2019.8189 31853294 PMC6909799

[22] Wu S, Hu R, He Z, et al. Effect of protocatechuic acid on growth performance, inflammatory status and immune indices in weaned piglets [J]. J Anim Sci. 2019;97:361–362. doi: 10.1093/jas/skz258.722

[23] Wang L, Hu R, Ma S, et al. Dihydroquercetin attenuated Prevotella copri-caused intestinal injury by modulating gut microbiota and bile acids in weaned piglets [j]. Anim Nutr. 2025;20:303–310. doi: 10.1016/j.aninu.2024.10.002 39995524 PMC11849659

[24] Hu R, Wu S, Li B, et al. Dietary ferulic acid and vanillic acid on inflammation, gut barrier function and growth performance in lipopolysaccharide-challenged piglets [J]. Anim Nutr. 2022;8(1):144–152. doi: 10.1016/j.aninu.2021.06.009 34977384 PMC8683658

[25] Yang X, Hu R, Yao L, et al. The role of uterus mitochondrial function in high-fat diet-related adverse pregnancy outcomes and protection by resveratrol [J]. Food Funct. 2024;15(9):4852–4861. doi: 10.1039/D4FO00671B 38573228

[26] Hu R, Yang X, Gong J, et al. Patterns of alteration in boar semen quality from 9 to 37 months old and improvement by protocatechuic acid [J]. J Anim Sci Biotechnol. 2024;15(1):78. doi: 10.1186/s40104-024-01031-6 38755656 PMC11100174

[27] Xie M, Chen W, Lai X, et al. Metabolic responses and their correlations with phytochelatins in Amaranthus hypochondriacus under cadmium stress [J]. Environ Pollut. 2019;252:1791–1800. doi: 10.1016/j.envpol.2019.06.103 31299508

[28] He K, Xiong J, Yang W, et al. Metagenome of gut microbiota provides a novel insight into the pathogenicity of Balantioides coli in weaned piglets [j]. Int J Mol Sci. 2023;24(13):10791. doi: 10.3390/ijms241310791 37445967 PMC10342044

[29] Zhou X, Liu Y, Xiong X, et al. Intestinal accumulation of microbiota-produced succinate caused by loss of microRNAs leads to diarrhea in weanling piglets [J]. Gut Microbes. 2022;14(1). doi: 10.1080/19490976.2022.2091369 PMC9235893 35758253

[30] Hao Q-Y, Yan J, Wei J-T, et al. Prevotella copri promotes vascular calcification via lipopolysaccharide through activation of NF-κB signaling pathway [j]. Gut Microbes. 2024;16(1):2351532. doi: 10.1080/19490976.2024.2351532 38727248 PMC11093026

[31] Dai Z, Deng K-L, Wang X-M, et al. Bidirectional effects of the tryptophan metabolite indole-3-acetaldehyde on colorectal cancer [j]. World J Gastrointest Oncol. 2024;16(6):2697–2715. doi: 10.4251/wjgo.v16.i6.2697 38994159 PMC11236226

[32] Karger KM, Ogilvie G, Tsai MY. A-326 argininosuccinic acid - is it routinely detectable in the urine of healthy individuals? [J] Clin Chem. 2023;69(Supplement_1). doi: 10.1093/clinchem/hvad097.288

[33] Lessa AYC, Edwinson A, Sato H, et al. Transcriptomic and metabolomic correlates of increased colonic permeability in postinfection irritable bowel syndrome [J]. Clin Gastroenterol Hepatol. 2025;23(4):632–643.e13. doi: 10.1016/j.cgh.2024.06.028 38987012 PMC11707044

[34] Wright DP, Rosendale DI, Robertson AM. Prevotella enzymes involved in mucin oligosaccharide degradation and evidence for a small operon of genes expressed during growth on mucin [J]. FEMS Microbiol Lett. 2000;190(1):73–79. doi: 10.1111/j.1574-6968.2000.tb09265.x 10981693

[35] Stettner N, Rosen C, Bernshtein B, et al. Induction of nitric-oxide metabolism in enterocytes alleviates colitis and inflammation-associated colon cancer [J]. Cell Rep. 2018;23(7):1962–1976. doi: 10.1016/j.celrep.2018.04.053 29768197 PMC5976577

[36] Gong R, He L, Zhou H, et al. Down-regulation of argininosuccinate lyase induces hepatoma cell apoptosis through activating Bax signaling pathway [J]. Genes Dis. 2019;6(3):296–303. doi: 10.1016/j.gendis.2018.11.003 32042869 PMC6997574

[37] Song Y, Li M, Liu J, et al. Screening study of hydroxytyrosol metabolites from in vitro fecal fermentation and their interaction with intestinal barrier repair receptor AhR [J]. J Food Sci. 2024;89(12):10134–10151. doi: 10.1111/1750-3841.17609 39686652 PMC11673453

[38] Chen Y, Wang Y, Fu Y, et al. Modulating AHR function offers exciting therapeutic potential in gut immunity and inflammation [J]. Cell Biosci. 2023;13(1). doi: 10.1186/s13578-023-01046-y PMC10182712 37179416

[39] Mayers JR, Varon J, Zhou RR, et al. Identification and targeting of microbial putrescine acetylation in bloodstream infections [J]. BioRxiv : Prepr Serv Biol. 2023 558834 doi:10.1101/2023.09.21.558834.

[40] Schirmer M, Strazar M, Avila-Pacheco J, et al. Linking microbial genes to plasma and stool metabolites uncovers host-microbial interactions underlying ulcerative colitis disease course [J]. Cell Host Microbe. 2024;32(2):209–226.e7. doi: 10.1016/j.chom.2023.12.013 38215740 PMC10923022

[41] Zhang Y, Mu T, Deng X, et al. New insights of biological functions of natural polyphenols in inflammatory intestinal diseases [J]. Int J Mol Sci. 2023;24(11):9581. doi: 10.3390/ijms24119581 37298531 PMC10253449

[42] Orlo E, Russo C, Nugnes R, et al. Natural methoxyphenol compounds: antimicrobial activity against foodborne pathogens and food spoilage bacteria, and role in antioxidant processes [J]. Foods. 2021;10(8):1807. doi: 10.3390/foods10081807 34441583 PMC8392586

[43] Zhang X, Li C, Li J, et al. Protective effects of protocatechuic acid on acute lung injury induced by lipopolysaccharide in mice via p38MAPK and NF-κB signal pathways [j]. Int Immunopharmacol. 2015;26(1):229–236. doi: 10.1016/j.intimp.2015.03.031 25841318

[44] Salama AAA, Elgohary R, Fahmy MI. Protocatechuic acid ameliorates lipopolysaccharide-induced kidney damage in mice via downregulation of TLR-4-mediated IKBKB/NF-κB and MAPK/Erk signaling pathways [J]. J Appl Toxicol. 2023;43(8):1119–1129. 36807594 10.1002/jat.4447

[45] Farombi EO, Adedara IA, Awoyemi OV, et al. Dietary protocatechuic acid ameliorates dextran sulphate sodium-induced ulcerative colitis and hepatotoxicity in rats. Food Funct. 2016;7(2):913–921. doi: 10.1039/C5FO01228G 26691887

[46] Peng Q, Ma Y, Wang Z, et al. Inhibition mechanism of different structural polyphenols against α-amylase studied by solid-state NMR and molecular docking [J]. Int J Biol Macromol. 2024;275:133757. doi: 10.1016/j.ijbiomac.2024.133757 38986997

[47] Vyas K, Prabaker S, Prabhu D, et al. Study of an inhibitory effect of plant polyphenolic compounds against digestive enzymes using bench-working experimental evidence predicted by molecular docking and dynamics [J]. Int J Biol Macromol. 2024;259:129222. doi: 10.1016/j.ijbiomac.2024.129222 38185307

[48] Marchese E, Cantafio MEG, Ambrosio FA, et al. New insights for polyphenolic compounds as naturally inspired proteasome inhibitors [J]. Pharmaceuticals. 2023;16(12):1712. doi: 10.3390/ph16121712 38139838 PMC10747119

[49] Gong J-T. Protocatechuic acid attenuated inflammation caused by *Prevotella copri* and its metabolites [data set]. Mendeley Data. 2025;V1. doi: 10.17632/hzcfbrxmcg.1 PMC12773465 41437510

