# Supplementary material for: Protocatechuic acid attenuated inflammation caused by Prevotella copri and its metabolites
Source: Virulence. 2025 Dec 23;17(1):2609387. doi: 10.1080/21505594.2025.2609387 (PMC12773465; doi:10.1080/21505594.2025.2609387)
Supplement: Supplementary data.docx [file KVIR_A_2609387_SM4265.docx]

**Supplemental Table 1.** Antibody information and incubation conditions

| **Antibodies** | **Cat. No.** | **Diluted ratio** | **Loading quantity of protein sample** | **Incubation conditions** | **Exposure time** |
| --- | --- | --- | --- | --- | --- |
| Phospho-NF-κB p65 (Ser536) | #3033, CST | 1:1000 | 40μg | 4 °C overnight | 6-12s |
| NF-κB p65 | #6956, CST | 1:1000 | 40μg | 4 °C overnight | 8-15s |
| IL-1β | A7F7-R, HUABIO | 1:1000 | 30μg | 4 °C overnight | 10-20s |
| IL-6 | BS62100, Bioworld | 1:1000 | 30μg | 4 °C overnight | 10-20s |
| COX-2 | #12282, Cell Signaling Technology | 1:1000 | 30μg | 4 °C overnight | 10-20s |
| Occludin | EPR20992, Abcam | 1:1000 | 50μg | 4 °C overnight | 80-100s |
| Claudin-1 | EPR9306, Abcam | 1:1000 | 50μg | 4 °C overnight | 80-100s |
| ZO-1 | HL1185, Abcam | 1:1000 | 50μg | 4 °C overnight | 100-120s |
| β-actin | #4970, CST | 1:1000 | 10μg | 4 °C overnight | 3-5s |
| HRP-linked Antibody | CST# 7074 | 1: 5,000 | - | Room temperature for 1 h |  |

**Supplemental Table 2. Composition and nutrient levels of the basal diet (air-dry basis, %)**

| Item | Content |
| --- | --- |
| Ingredients |  |
| Wheat for pigs | 37.16 |
| Suckling pig corn | 15.00 |
| Low protein whey powder | 9.00 |
| Expanded soybean | 7.00 |
| Hamlet protein HP300 | 6.50 |
| Flour | 5.00 |
| Soybean meal (46 %) | 5.00 |
| Fermented soybean meal | 5.00 |
| Soybean oil | 2.35 |
| Biological feed | 2.00 |
| Calcium hydrogen phosphate | 1.87 |
| Lysine (95 %) | 0.95 |
| Vitamin premixed core material^1^ | 0.60 |
| Stone powder | 0.50 |
| Trace mineral element premixed core material | 0.40 |
| Sodium chloride | 0.30 |
| Acyclol (phosphoric acid, lactic acid) | 0.30 |
| Methionine (99 %) | 0.23 |
| Threonine (98 %) | 0.22 |
| Acridine (benzoic acid, lactic acid) | 0.20 |
| Zinc oxide (95 %) | 0.19 |
| Zinc oxide (95 %) | 0.12 |
| Choline chloride (60 %) | 0.08 |
| Tryptophan (98 %) | 0.06 |
| Total | 100.00 |
| Energy and nutrient levels |  |
| Digestible energy, MJ/kg | 14.58 |
| Crude protein | 19.22 |
| Crude fat | 5.43 |
| Crude fiber | 2.44 |
| Crude ash | 5.85 |
| Neutral detergent fiber | 10.22 |
| Acid detergent fiber | 3.64 |
| Lysine | 1.41 |
| Calcium | 0.87 |
| Total phosphorus | 0.76 |

^1^The vitamin premixed core material provided per kilogram of feed: vitamin A, 10,500 IU; vitamin D_3_, 3,300 IU; vitamin E, 22.5 IU; vitamin K_3_, 3 mg; vitamin B_1_, 3 mg; vitamin B_2_, 7.5 mg; vitamin B_6_, 4.5 mg; vitamin B_12_, 0.03 mg; niacin, 30 mg; pantothenate, 15 mg; folic acid, 1.5 mg; biotin, 0.12 mg.

**Supplemental Table 3.** GenBan accession numbers, sequences of forwardand reverseprimers, and fragment·sizes used for Real-Time PCR

| **Target** | **GeneBank number** | **Primer sequence** | **Size, bp** |
| --- | --- | --- | --- |
| β-actin | XM_  003124280.5 | F: CTATTGGCAACGAGCGGTTCC | 150 |
|  |  | R: GCACTGTGTTGGCATAGAGGTC |  |
| IL-2 | NM_213861.1 | F: CACAAAGAAACAACTGGAGCCATTG | 128 |
|  |  | R: GCATCCTGGAGAGATCAGCATTC |  |
| IL-6 | NM_214399.1 | F: TTCAGTCCAGTCGCCTTCT | 97 |
|  |  | R: TGGCATCACCTTTGGCATCTTC |  |
| IL-8 | NM_213867.1 | F: AGCTTGTCAATGGAAAAG AGGTCTG | 101 |
|  |  | R: CTGTTGTTGTTGCTTCTCAGTTCTC |  |
| IL-10 | NM_214041.1 | F: CCGAAGGCAGAGAGTGATGGG | 111 |
|  |  | R: ACAGGGCAGAAATTGATGACAGC |  |


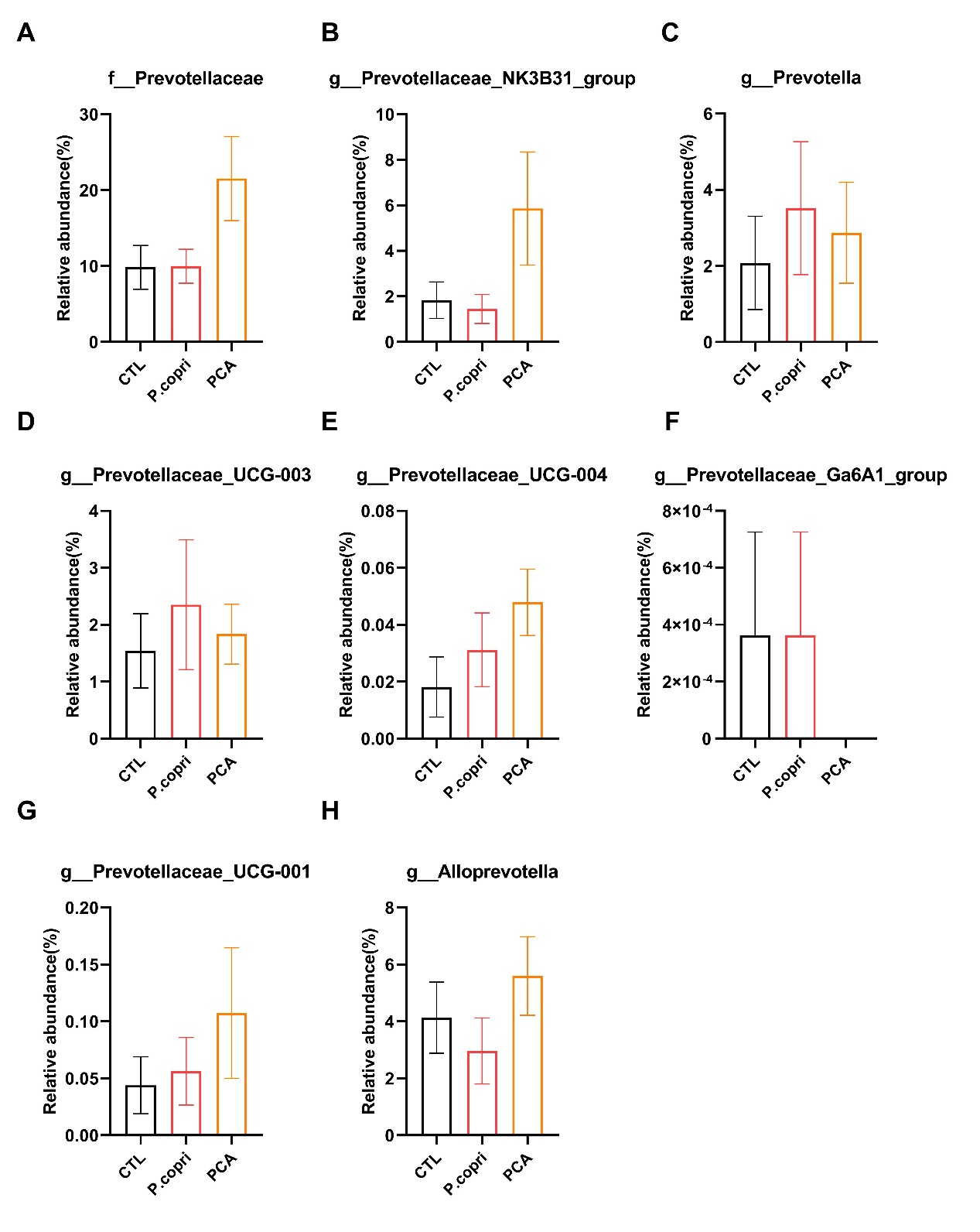


**Supplementary figure 1.** Relative abundance of *Prevotella* in the gut of piglet. Differences in the *Prevotellaceae* in the gut of mice**(A)** . Differences in four different *Prevotella* genera in the gut of mice **(B-H)**. Data are shown as the means ± SEMs (*n*=6), * *P＜0.05*, ** *P＜0.01*.
